# Effects of Non-Coding RNA on Regulatory T Cells and Implications for Treatment of Immunological Diseases

**DOI:** 10.3389/fimmu.2020.612060

**Published:** 2020-11-20

**Authors:** Yuanhanyu Luo, Honglin Wang

**Affiliations:** Institute of Translational Medicine, Shanghai General Hospital, Shanghai Jiao Tong University School of Medicine (SJTU-SM), Shanghai, China

**Keywords:** regulatory T cells, non-coding RNAs, post-transcriptional regulation, immunological tolerance, immunological disorders

## Abstract

Regulatory T cells (Tregs) are essential for regulating immune reactions and maintaining immune homeostasis. Non-coding RNAs (ncRNAs), including microRNAs and long non-coding RNAs, usually do not encode proteins but regulate intracellular biological processes at post-transcriptional levels. These ncRNAs have been demonstrated as key post-transcriptional regulators in the commitment of Tregs lineage and the plasticity of Tregs function. These ncRNAs can further be manipulated to benefit human immunological disorders caused by Tregs dysfunction. This review summarizes the effects of ncRNAs on Tregs and their potentials to be targets or approaches for the treatment of immunological diseases involving Tregs.

## Introduction

Regulatory T cells (Tregs), recognized as a specialized subset of CD4^+^ T cells, are essential mediators in maintaining immune tolerance by suppressing immune reactions (1). Three subsets of CD4^+^ Tregs have been characterized, namely thymus-derived Treg (tTreg) and the peripherally induced Treg (pTreg) developed from mature CD4^+^ conventional T cells outside of the thymus and *in vitro*-induced regulatory T (iTreg) cells (2). Forkhead box P3 (Foxp3) has been identified as a master transcriptional factor in maintaining differentiation and suppressive function of Tregs (3). Downregulated Foxp3 expression commonly causes immune dysregulation, which elicits autoimmune diseases (4). Mutation in the Foxp3 gene impairs Tregs development and function, which further causes immune dysregulation polyendocrinopathy and enteropathy X-linked syndrome along with other grievous autoimmune diseases. Likewise, Foxp3-depleted or Foxp3-mutated mice have Tregs deficiency and development of autoimmunity (3, 5, 6).

Transcriptome and proteome studies of Tregs unveil post-transcriptional regulators manipulating Tregs development and function, which highlights the importance of non-coding RNA regulation (7). Non-coding RNAs (ncRNAs), comprising microRNA and long non-coding RNAs etc., are not able to encode proteins but still contain important information (8). MicroRNAs (miRNA) commonly contain ~22 nucleotides, which have function in RNA silencing and post-transcriptional regulation of gene expression. Their altered expression is observed in specific biological and pathological processes. MiRNAs bind to their respective target RNAs either in the translational regions or in the 3’ untranslated regions (3’UTR). Particularly, miRNAs modifies mouse Tregs development, homeostasis, and normal function but in humans, their roles remain elusive (9). miRNAs play a crucial role in preserving Tregs suppressive function through Dicer-dependent pathway (10). LncRNAs harbor an abundant proportion of the non-coding transcriptomes. However, lncRNAs characteristics, function mechanisms in Tregs still remain obscure.

In this review, we firstly introduce the biogenesis of non-coding RNAs and exertion of suppressive function in Tregs. Then we summarize ncRNA-mediated post-transcriptional modifications of Tregs and discuss the potential therapeutic strategies for human immunological disorders with dysfunctional Tregs involved.

CD4+CD25+Foxp3+ regulatory T cells (Tregs) play a crucial role in maintaining immune homeostasis and self-tolerance through suppressing excess immune responses. Foxp3, the master transcription factor, is highly expressed in Tregs. Researchers found that Foxp3 programs development and function of Tregs since ectopic Foxp3 expression confers suppressor function in Tregs (11). Targeted deletion of mice Foxp3 resulted in severe lymphoproliferative autoimmune syndrome with evidently enlarged spleen and lymph nodes. Mutations of FOXP3 in human trigger several immune disorders, polyendocrinopathy, enteropathy and X-linked syndrome (IPEX) (12).

*In vitro* model systems identify the mechanisms by Tregs to suppress a large range of target cell types. These mechanisms can be divided into those that target responder Foxp3^-^ T cells and those that initially target antigen-presenting cells. Tregs secret multiple suppressive cytokines like IL-10, TGF-β and IL-35 to regulate the activity of effector T cells. Tregs also overexpress CD25 (IL-2 receptor) to deprive local IL-2 resulting in the suppression of effector T cells. Activated Tregs may directly kill effector cells in a perforin-dependent and granzyme-mediated manner (13). On the other hand, Tregs may affect the function of APC indirectly inhibiting the activation of effector T cells. CTLA-4 on Tregs surface interacts with CD80 or CD86 on dendritic cells to downregulate costiulation, which is important for Tregs to exert their suppressive function. One other antigen on Tregs surface that affect Tregs function is LAG-3. LAG-3 binds to MHC class II molecules on immature DCs surface resulting in downregulated costimulatory capacity (14).

*In vivo*, Tregs residing in visceral adipose tissue (VAT) display distinct suppressive features in homeostasis maintenance (14). VAT-Tregs highly express the enzyme hydroxyprostaglandin dehydrogenase (HPGD), which converts PGE_2_ into the metabolite 15-keto PGE_2_. Once HPGD expression is promoted by PPARγ, the generation of 15-keto PGE_2_ inhibited other T cells activation and proliferation (14). Skeletal muscle Tregs and colonic Tregs also play an important part in local tissue homeostasis but their detailed mechanisms still need more research (14).

## Biogenesis of Non-Coding RNAS to Regulate the Post-Transcription Modification

The RNA world is divided into two parts——RNAs with coding potential and those without, also referred to as non-coding RNAs (ncRNAs). Though ncRNAs seem not to encode protein, they play an essential role in cell development and physiology. Also, the huge amount of ncRNAs form a complex regulation network in cells. ncRNA can be classified into two subclasses roughly by sequence size: small non-coding RNAs (ncRNAs smaller than 200 nucleotides) and long non-coding RNAs (lncRNAs, longer than 200 nt). Herein, we introduce biogenesis and mechanisms of microRNAs (miRNAs) and long non-coding RNAs in general (15, 16).

MicroRNAs (miRNAs) are commonly generated from transcriptional units or non-coding regions (17). In the beginning, miRNA genes are transcribed by RNA polymerase II into primary miRNA transcripts (pri-miRNA). pri-miRNA is relatively long with a 5’7-methyl guanosine cap and a 3’ poly-adenylated hairpin structure (18). Pri-miRNA processing needs two ribonuclease III (RNase III) enzymes——Drosha and Dicer. Firstly, in nucleus pri-miRNA transcripts are cleaved into 60-70 nucleotides precursor miRNA (pre-miRNA) by Drosha and its cofactor DiGeorge syndrome critical region gene 8 (DGCR8) forming a small hairpin structure (19, 20). Then pre-miRNAs are transported into the cytoplasm *via* Exportin 5 and be cut by another RNase III enzyme, Dicer into several small double-stranded RNA which is about 19–22 nucleotides long (21–23). One strand of the duplex is loaded into the RNA-induced silencing complex (RISC) while the other strand is degraded. RISC which consists of Dicer, TRBP, and the Argonaute proteins recognize target mRNAs in their 3’UTR to promote target mRNA degradation (24). According to such a unique mechanism, miRNA is able to specifically affect many key genes in the signal pathway.

Long non-coding RNAs (LncRNAs) are relatively less studied in ncRNA species due to their sequence heterogeneity. LncRNAs are transcribed by Pol II or Pol III and then lncRNAs are processed with m7G cap and polyadenylation (9). Subsequently, they are released into cytoplasm for further processing and stabilization to form a higher-order structure. LncRNAs exert their regulatory function through acting as sponges for miRNAs or degrading mRNAs in the cytoplasm. However, the detailed process of lncRNAs biogenesis and cofactors to ensure lncRNAs function remain elusive. It will be a challenging discovery for researchers to explore further (9).

## Non-Coding RNA Modifications Involved in Treg Lineage Commitment and Suppressive Function

### MicroRNAs

**Figure 1:**
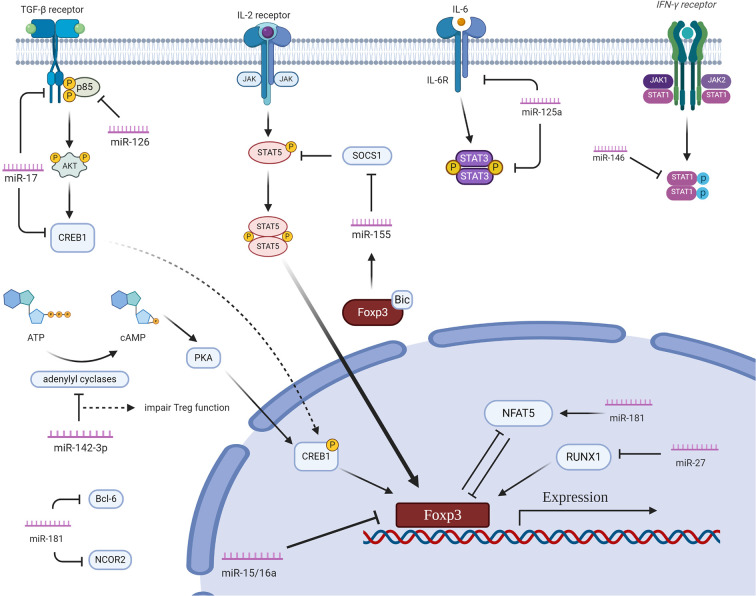
The regulatory network of miRNAs in Tregs. miR15a/16 directly binds FOXP3, which inhibits Treg differentiation. miR-155 compromises IL-2 production *via* specifically targeting SOCS1. miR-146a downregulates Stat1 to alleviate IFN-γ signaling. miR-17 directly targtes TGF-β receptor II and cAMP-responsive element binding protein 1 thus negatively regulating Treg differentiation. miR-126 targets the 3’UTR of p85β, an important regulatory subunit of PI3K, reducing the expression of Foxp3. miR-10a directly targets Bcl-6 and Ncor2. miR-142-3p binds with adenylyl cyclases, which impairs the suppressive effect of Tregs. miR-125a regulate the function of Tregs by targeting Stat3. miR-181a directly inhibits NFAT5 which is an important transcriptional factor for Foxp3. miR-27 downregulates RUNX1, which impairs the differentiation of Tregs. Created with BioRender.com.

miRNAs play important regulatory roles in Tregs by pairing to target mRNAs. MiRNAs finely manage gene expression by directly interacting with 3’untranslated regions or other gene regions. MiRNAs form a complex network of regulation in Tregs (**Figure 1**).

miR15a/16, as studies showed in cord blood (CB) Tregs, were negatively associated with FOXP3 expression (25). miR15a/16 expression were significantly lower in CB Tregs when compared with conventional T cells. Overexpression of miR15a/16 in CB Tregs was demonstrated to inhibit normal FOXP3 transcription as well as CTLA4, a Foxp3-dependent gene in Tregs (25). On the other side, knockdown of miR15a/16 in CB T cells had been demonstrated to promote expression of FOXP3 and CTLA4, indicating that miR15a/16 play an important role in regulating Treg plasticity. miR15a/16 were reported to directly target specific regions of FOXP3 3’UTR. Thus, miR15a/16 are key regulators of Treg ratio at the post-transcriptional level (25).

miR-155 is highly expressed in Tregs. Genome-wide analysis reveals that Foxp3 bind to the B cell integration cluster (Bic) which harbors the primary miR-155 transcript (26, 27). Kohlhaas et al. reported that miR-155-deficient mice exhibited decreased numbers of Tregs in both thymi and spleens, although the suppressive function of Tregs *in vitro* remained unaffected (28). A high amount of miR-155 was maintained by Foxp3 expression in Tregs and sustained in a Foxp3-dependent pattern. miR-155 specifically targets suppressor of cytokine signaling 1 (SOCS1) to facilitate Tregs homeostasis. The reduced numbers of Tregs in miR155-deficient mice were in line with that in SOCS1-transgenic mice (29). miR-155 deletion in CD4^+^ T cells compromised IL-2 production, which suggests that miR-155 likely regulates IL-2-associated Tregs homeostasis *via* both cell-intrinsic and cell-extrinsic mechanisms. Reduced STAT5 phosphorylation in miR155-deficient Tregs offered an alternative explanation for decreased Treg numbers in miR155-deficient mice (29).

miR-146 is prominently expressed in Tregs. Increased numbers of Foxp3^+^ Tregs in the periphery were observed in miR-146a-deficient mice. Moreover, miR-146a-deficient Tregs were insufficient to restrain the activation of T effector cells (30). miR-146a deficiency lead to development and exhibition of a serious lymphoproliferative and myeloproliferative syndrome in mice at 6 months of age. Stat1 is the target of miR-146a in human peripheral blood mononuclear cells (hPBMCs) since the 3’ UTR of the human *Stat1* gene perfectly matches the miR-146a sequence. Phosphorylated Stat1 is increased in miR-146a-deficient Tregs. Stat1 is a key transcription factor downstream of IFN-γ receptor signaling. miR-146a expression in Tregs is crucial to control Th1 immune responses mediated by IFN-γ *via* targeting Stat1 (30). Nevertheless, the role of miR-146a in autoimmune diseases remains to be determined using conditional miR-146a-deficient mice, since miR-146a-deficient myeloid cells promotes Tregs dysfunction and autoimmunity (31, 32).

miR-17-92 cluster is encoded by a single polycistronic host gene which produces 6 individual mature miRNAs. These miRNAs can be classified into 3 families based on sequence homology: the miR-17 family (miR-17, miR-20, and miR-18a), the miR-19 family (miR-19a and miR-19b), and the miR-25 family (miR-92a). miR-17 and miR-19b inhibit iTreg differentiation. Consistently, elimination of the miR-17-92 cluster in CD4^+^ T cells promoted Foxp3 expression. miR-17 directly targtes TGF-β receptor II (Tgfbr2) and cAMP-responsive element binding protein 1 (Creb1), both of which drive Treg differentiation (33). In addition, miR-17-92 engaged in costimulatory network of CD28, which is important for thymic development and survival of Tregs. CD28-mediated costimulation is crucial in the production of IL-10 in Tregs to ensure Treg immunosuppressive function. The frequency of IL-10 producing Tregs is remarkably reduced in miR-17-92 depleted Tregs demonstrating that miR-17-92’s essential role in regulating Tregs normal function (34). Conclusively, miR-17-92 plays an important role in Tregs differentiation by interacting with certain important proteins like TGF-βr and CREB1 and regulating IL-10 secretion in Tregs.

iTregs express high levels of miR-126. Disturbance of miR-126 using its antisense oligonucleotides (ASO) significantly inhibits the induction of Tregs. *In vivo* miR-126 abrogation dramatically downregulates the expression of Foxp3 in Tregs as well as CTLA-4 and glucocorticoid induced tumor necrosis factor receptor (GITR) (35). miR-126 directly targets the 3’UTR of p85β, which is an important regulatory subunit of PI3K involved in PI3K/Akt pathway (36). miR-126 ASO significantly elevate the expression of p85β, contributing to the activation of PI3K/Akt pathway in Tregs, the reduced expression of Foxp3, and the impaired suppressive function of Tregs (37, 38).

miR-10a is a Treg-specific miRNA (39, 40). miR-10a gets involved in long-term maintenance of pTreg stability. Combination of all-trans Retinoid acid (ATRA) and TGF-β dramatically boosts miR-10a in a dose-dependent manner, leading to optimal induction of miR-10a in Tregs. miR-10a targets Bcl-6 and Ncor2 through binding to their 3’UTRs (41). In Peyer’s patches (PP), Tregs can be converted to T follicular helper (Tfh) cells, which is limited by miR-10a *via* dampening Bcl-6 expression (41). Intriguingly, miR-10a is dispensable for the direct regulation of Foxp3 or other related factors in iTregs. In mice deficient of miR-10a, neither the number nor function of Tregs was altered, when treated with RA and TGF-β (41).

miR-142-3p regulates Tregs function in a unique way. Naturally, Tregs maintain high levels of cAMP and transfer cAMP to conventional T cells through gap junction channels to facilitate immunomodulation. Intracellular adenylyl cyclases (ACs) are key enzymes for cAMP generation. miR-142-3p directly targets the 3’UTR of AC9 in Tregs. Therefore, miR-142-3p restricts the generation of cAMP and impairs the suppressive function of Tregs. Blockage of miR-142-3p with inhibitors restores the cytokine production and suppressive function of Tregs. Foxp3 downregulates miR-142-3p to maintain the activity of AC9 and the production of cAMP. The downregulation of miR-142-3p might get involved in the mechanisms of which Foxp3 upregulates the expression of Treg-associated genes in the Tregs (42).

miR-125a is downregulated in peripheral CD4^+^ T cells of several human auto-inflammatory diseases including systemic lupus erythematosus and Crohn’s disease as well as relevant mouse models (43). In miR-125a knockout mice, proportions of CD4^+^ and CD8^+^ T cells in lymph nodes and spleens were not affected while the percentages and absolute numbers of Tregs suffered a significant decline (43). miR-125a deficiency impaired the ability of T cells to differentiate into Tregs *in vitro*. Mechanistically, miR-125a directly represses several targets which inhibit Tregs differentiation, including Stat3, IL-13, IFN-γ and IL-6R (44). miR-125a also directly modulates CCR2 expression of Tregs in pancreatic draining lymph nodes, which limits Tregs migration and affects maintenance of immune tolerance in the pancreas (45).

Expression levels of IL-10 and TGF-β are higher in miR-181a-overexpressing CD4^+^ T cells than controls. Although miR-181 does not influence Treg differentiation, it elevates IL-10 and TGF-β *via* PI3K/Akt and SIRT1 pathways to enhance the suppressive function of Tregs (46). On the other hand, another study showed that enhancement of miR-181 up-regulated nuclear factor of activated T cells 5 (NFAT5), restraining Foxp3^+^ Tregs induction *in vitro*. This mechanism indicates that inhibited production of miR-181 promotes Tregs induction in mouse autoimmune model and alleviates murine islet autoimmunity *in vivo* (47). These results showed that miR-181a may play pro-inflammatory or anti-inflammatory roles. Also, specific targeting miR-181a sheds light on medicine development aiming at limiting islet autoimmunity or other autoimmune diseases (47).

miR-27 belongs to the miR-23~27~24 family and is upregulated in T cells isolated from patients with multiple sclerosis (MS) (48). miR-27 directly repressed B lymphoma Mo-MLV insertion region 1 homolog (Bmi-1), whose protein stabilizes the Th2 transcription factor, GATA3. Thus, forced miR-27 overexpression represses Bmi-1, facilitates GATA3 degradation and reduces Th2 responses. It triggers pathogenic Th1 responses in mice with T cell miR-27 overexpression (49). On the other hand, Forced miR-27 overexpression in murine T cells severely impaired Tregs generation by directly targeting runt-related transcription factor 1 (RUNX1), SMAD2/3, and c-Rel, which are known as members of the NF-κB family and play indispensable roles in initiating Foxp3 transcription. In addition, miR-27 overexpression suppresses Tregs function *via* targeting IL-10 (50) (**Table 1**).

**Table 1:** Characteristics of MiRNAs in Tregs.

MiRNAs	Impact on Tregs	Targets	Human immunological diseases or animal models
miR15a/16	Impair suppressive function	Foxp3	Human cord blood
miR-155	Maintain suppressive function	SOCS1	Mice and Kawasaki diseases
miR-146	Promote suppressive function	IFN-γ signaling	Mice
miR-17-92	Promote Tregs differentiation	TGF-β signaling pathway	Mice
miR-126	Promote suppressive function	PI3K/Akt pathway	Mice
miR-10a	Maintain Tregs stability	Bcl-6 and Ncor2	Mice
miR-142-3p	Maintain Tregs suppressive function	Adenylyl cyclases	Mice
miR-125a	Inhibit Tregs differentiation	Stat3, IL-13	Lupus erythematosus and Crohn’s disease as well as mice
miR-181a	Enhance the suppressive function	NFAT5	Islet autoimmunity in mice
miR-27	Impair Tregs generation	RUNX1, SMAD2/3 and c-Rel	Human multiple sclerosis

### LncRNAs

LncRNAs are commonly described to be longer than ~200 nucleotides with the ability to modulate chromatin structure and regulate gene expression at both transcriptional and post-transcriptional levels.

Hepatocellular carcinoma up-regulated lncRNA (HULC) contributes to Tregs differentiation by targeting p18 and is engaged in hepatocellular carcinoma (HCC) development and progression (51).

lncRNA SNHG1 promotes Tregs differentiation and drives the progression of colorectal carcinoma. SNHG1 binds to miR-448, the latter directly targets Indoleamine 2,3-dioxygenase (IDO), which is a inducer of Tregs. Thus, SNHG1 induces Tregs differentiation and facilitates immune escape through suppressing miR-448 function (52, 53).

Foxp3 long intergenic noncoding RNA (Flicr) impairs Tregs differentiation *via* reducing the chromatin accessibility in the Foxp3 locus under low IL-2 levels. Once Flicr is deleted, Treg restores stabilized level of Foxp3 levels, suggesting that Flicr modulation is likely to serve as a switch to enhance or inhibit Treg suppressive function (54).

## Implications for the Treatment of Human Immunological Diseases

Kawasaki disease (KD) is an acute systemic inflammatory disease, which subjects children to coronary artery aneurysms, myocardial infarction or even sudden death (55). KD onset involves immune dysfunction with descending Treg proportions and decreased expression of Foxp3. As previously mentioned, miR-155 is required for normal Tregs frequency and function. KD patients show decreased miR-155, which is a factor contributing to Tregs deficiency and KD progression (56). Restoring miR-155 expression in KD patients is a promising strategy.

MiR-21 has been demonstrated to play an important role in regulating the Th17/Treg balance. miR-21 upregulates Foxp3 and promotes Tregs differentiation. miR-21 alleviates rheumatoid arthritis (RA) *via* boosting the immunomodulatory properties of Tregs (57).

miR-31 was found to directly target Foxp3 and lentiviral transduction of miR-31 evidently reduced Foxp3 expression (8). In another study, miR-31 deficiency boosted Tregs differentiation through targeting protein phosphatase 6c (Ppp6c), which then led to decreased blood pressure, relieved vascular damage and alleviated hypertension pathology. Studies also showed that miR-31 inhibited pTreg generation *via* directly targeting Gprc5a (58).

miR-210 has been revealed to be critical for psoriasis pathology in several aspects (59). miR-210 expression is upregulated in peripheral blood CD4^+^ T cells as well as in the skin lesions of patients with psoriasis. Functionally, overexpression of miR-210 aggravates psoriasis and deletion of miR-210 inhibits psoriasis development. Thus, miR-210 proves a promising drug target for the treatment of psoriais (60).

miR-214, conventionally considered as an oncogenic miRNA, promotes human ovarian cancer through directly targeting phosphatase and tensin homolog (PTEN). Secreted miR-214 from Lewis lung carcinoma (LLC) cells is a strong inducer of Tregs expansion both *in vitro* and *in vivo*. Consistently, blockage of miR-214 using ASO undermines Tregs induction and thus inhibits tumor growth. Importantly, cell-derived microvesicles (MVs) were employed to deliver miR-214 ASO to human peripheral CD4^+^ T cells, which increases PTEN expression and inhibits Tregs expansion. This may offer an innovative avenue to tumor therapy (61).

## Conclusion

Non-coding RNAs play an important role in regulating Tregs differentiation and function. We conclude a range of miRNAs and long-coding RNAs and reveal their effects on Tregs. These miRNAs provide a pool of targets for treatments of Tregs-mediated autoimmune disease. However, this field needs more exploration. The roles of several miRNA in Tregs and other T cell subsets are still elusive both *in vitro* and *in vivo.* Therefore, what we need is the big picture of interaction between miRNAs in Tregs, which will be helpful for understanding underlying mechanisms and discovering potential therapies.

## Author Contributions

YL wrote the manuscript. HW and YL designed the structure and content of this review. All authors contributed to the article and approved the submitted version.

## Funding

This work is supported by grants from the National Natural Science Foundation of China (81930088, 81725018, 81703118 and 81803123), Shanghai Collaborative Innovation Center for Translational Medicine (TM201925), Innovative Research Team of High-Level Local Universities in Shanghai.

## Conflict of Interest

The authors declare that the research was conducted in the absence of any commercial or financial relationships that could be construed as a potential conflict of interest.
